# Distribution of Selenium and Oxidative Stress in Breast Tumor-Bearing Mice

**DOI:** 10.3390/nu5020594

**Published:** 2013-02-20

**Authors:** Chih-Hung Guo, Simon Hsia, Pei-Chung Chen

**Affiliations:** Micro-Nutrition & Biomedical Nutrition Labs, Institute of Biomedical Nutrition, Hung Kuang University, Taichung 433, Taiwan; E-Mails: eillyguo@sunride.hk.edu.tw (C.-H.G.); dr.simon.hsia@gmail.com (S.H.)

**Keywords:** selenium, oxidative stress, breast tumor, mice

## Abstract

The present study investigated the effects of breast tumors on the blood and tissue distribution of essential trace mineral selenium (Se), and oxidative stress status of mice. Female 10-week-old BALB/cByJNarl mice were randomly assigned into control (CNL) and breast tumor-bearing (TB) groups. TB mice were injected subcutaneously into the right hind thigh with 5 × 10^6^ EMT6 mouse mammary tumor cells. After 22 days, we measured Se concentrations, Se-dependent glutathione peroxidase (GPx) activities, and malondialdehyde (MDA) products (indicator of oxidative stress) in plasma, various tissues, and plasma vascular endothelial growth factor (VEGF) concentrations. There were no significant differences in body weights and daily intake between both groups. Compared with the CNL group, TB mice have decreases in plasma Se concentrations and GPx activities, as well as higher plasma VEGF and MDA concentrations. Plasma Se concentrations were also negatively correlated with plasma MDA and VEGF concentrations. Furthermore, tissue Se concentrations and GPx activities in TB animals were lower; whereas the MDA concentrations higher in various tissues including liver, kidney, brain, lung, spleen, and thymic tissues. In conclusion, disruption of Se homeostasis critically reflects oxidative stress in target tissues, thus may increase the risk for progression of breast cancer and metastasis.

## 1. Introduction

Breast cancer is the most commonly diagnosed cancer and the leading cause of cancer death among women worldwide [1]. It is estimated that more than 1.1 million women will be newly diagnosed with breast cancer, and more than 410,000 die from the disease annually [2]. In Taiwan, the incidence of this disease has increased approximately four-fold over the past twenty years. Further, breast cancer has become the most common female cancer and the fourth most common cause of cancer-related death [3]. It is important to clarify the risk factors contributing to breast cancer susceptibility and development, although several pathways, including glutamine metabolism, have been shown to be involved in breast cancer proliferation [4].

The major risk factors associated with breast cancer include age, being overweight, early menarche, late menopause, first pregnancy at late age, the use of postmenopausal hormones such as estrogen and progesterone, and the presence of an inherited mutation in the *BRCA1* or *BRCA2* breast genes [5]. Additionally, oxidative stress has been linked to breast cancer risk [6]. Oxidative stress causes DNA damage, which if unrepaired can lead to mutations in tumor suppressor genes [7]. Oxidative stress stimulates the over-expression of vascular endothelial growth factor (VEGF), an important factor for tumor angiogenesis and neovascularization [8]. Furthermore, cumulative evidence suggests that oxidative stress directly contributes to neoplastic progression and metastasis [9]. Metastasis is a major cause of death; in particular, breast cancer can metastasize to the lung, brain, liver, kidney, and bones [10]. Thus, alleviating oxidative stress in target tissues may attenuate metastatic potential of tumors [11].

Selenium (Se) is well known as an essential trace mineral and an essential cofactor for glutathione peroxidases (GPx), selenoprotein P, and thioredoxin reductase, which are involved in scavenging free radicals and maintaining the redox balance [12]. Growing evidence indicated that Se protects mammary epithelial cells from oxidative DNA damage [13,14], inhibits the initiation phase of carcinogenesis, stimulates DNA repair, regulates apoptosis, and prevents cells from angiogenesis [15,16]. Furthermore, the relationship between the Se status and breast cancer risk has been documented from clinical observations. Compared to healthy subjects, patients with cancer exhibit markedly lower plasma concentrations of Se and GPx activity [17,18]. Decreased Se intake status and lower toenail Se levels were associated with breast cancer risk in previous research [19,20]. *BRCA1* plays an important role in repair of oxidative DNA damage [21]; toenail Se concentrations were inversely associated with levels of chromosomal damage and oxidative DNA damage in BRCA1 mutation carriers [22]. Thus, disturbances in Se homeostasis aggravate oxidative stress and thus may stimulate tumor progression and metastasis [23]. Recent studies have shown the effects of Se on the anti-carcinogenic mechanisms; however, there is limited information regarding the distribution of Se in various tissues with cancer.

The purpose of the present preliminary investigation was to examine the effects of breast tumor on the blood and tissue distribution of Se, and oxidative stress status in mice.

## 2. Experimental Section

### 2.1. Animals

Sexually mature female BALB/cByJNarl mice were obtained from the National Laboratory Animal Breeding Research Centre (LABRC, Taiwan) and maintained under specific pathogen-free condition. Animals were fed lab chow (Ralston Purina, 5001, St. Louis, MO, USA) and distilled deionized water (18.3 MΩ cm resistance, Milli-Q, Millipore, Bedford, MA, USA) *ad libitum*, and the standard condition in the animal rooms was maintained at a 12-h light/dark cycle with a room temperature of 24 ± 1 °C. The experiment protocol had the approval from the institutional animal care committee of Hung Kuang University.

### 2.2. Implantation of Tumor Cells

EMT6, a mouse mammary tumor cell line that has been utilized to observe local tumor growth at the primary site and pulmonary metastasis [24]. EMT6 (CRL-2755), was purchased from the American Type Culture Collection (Rockville, MD, USA) and cultured in Waymouth’s MB 752/1 medium (GIBCO) with 2 mM L-glutamine. Cells were then maintained in medium, supplemented with 15% (v/v) heat-inactivated fetal bovine serum (Invitrogen, Carlsbad, CA, USA), at 37 °C in 5% CO_2_ humidified atmosphere.

After two weeks of acclimation, animals (aged ten weeks) were randomly assigned into two experimental groups, which were designated as control (CNL, *n* = 10) and breast tumor-bearing (TB, *n* = 10) groups. For inoculation into animals, cells were washed with PBS, incubated in PBS-EDTA for 10 min, and then re-suspended in PBS prior to injection. In a 22-days experiment, about 5 × 10^6^ EMT6 cells suspension in 300 μL sterile PBS and injected subcutaneously in the right hind thigh of each mouse. At the end of study, animals were sacrificed and blood drawn in the morning after 12 h of fasting, plasma was separated into metal-free plastic tubes, along with primary tumors, liver, kidney, lung, brain, spleen, thymus, and mammary tissues which were quickly excised, frozen in liquid nitrogen, and stored at −80 °C until needed. The tumor size was recorded using digital calipers; the volume of tumor was calculated using the formula (X^2^Y)/2, where X and Y are the short and long diameters, respectively.

### 2.3. Se Contents

Atomic absorption spectrophotometry (932AA, GBC, Melbourne, Australia) with the accessory hydride generation system (HG 3000, GBC, Melbourne, Australia) was used for determining Se concentrations. Samples were digested for a total of 10.5 h with an initial temperature of 60 °C for 1–1.5 h, followed by increasing temperatures by 20 °C increments and finally heated up to 225 °C for 2 h in a mixture of 3.2 mL nitric acid (^16^N), and 0.8 mL concentrated perchloric acid to convert all Se species to selenate [25]. The reduction of selenate was completed within 30 min at a block temperature of 120 °C [26]. Accuracy of the methods was confirmed by comparing to serum (level 2, NO0371) reference materials (Seronorm, Nycomed, Oslo, Norway). The intra- and inter-assay CVs (coefficient of variation) were 2.9% and 3.5%, respectively.

### 2.4. MDA Production

Malondialdehyde (MDA) concentrations, a metabolite of polyunsaturated fatty acids, were assessed as a marker for lipid peroxidation. Briefly, the erythrocyte or supernatant from tissue homogenates was mixed with 3% SDS, 0.1 N HCl, 10% phosphtungstic acid and 0.7% thiobarbituric acid, and then incubated at 95 °C for 60 min. The *n*-butanol was added and the mixture was shaken vigorously. After centrifugation at 12,000× *g* at 4 °C for 15 min, the thiobarbituric acid-reactive substances in the *n*-butanol layer were taken for measured with Wallac 1420 multilabel counter Victor 3 (PerkinElmer, Turku, Finland) using 530 nm with 485 nm excitation. The MDA levels were calculated using the 1,1,3,3-tetra-ethoxypropane as standards. In addition, protein concentrations were measured using the Coomassie protein assay (Pierce, Rockford, IL, USA) with bovine serum albumin as standard.

### 2.5. Antioxidant Enzyme Activities

GPx activity was determined using a commercial kit supplied by Cayman Chemical (cat #703102). Oxidized glutathione (GSSG) produced from reducing reactive oxygen species was recycled by NADPH and glutathione reductase (GR) to reduced glutathione (GSH). Therefore, the rate of NADPH consumption was utilized as a measurement of the rate of GSSG formation. The plasma or supernatants from tissue homogenates were mixed with the stock solution containing NADPH, GSH and excess GR, and incubated at 37 °C for 5 min, followed by addition of 20 μL of cumene hydroperoxide as a substrate. The GPx activities were expressed as nmol NADPH oxidized/min/mL (mg protein).

### 2.6. Circulating VEGF Levels

Plasma VEGF was measured using the Bio-Plex Pro cytokine set kit, involving incubation with antibody-conjugated beads against mouse VEGF and assays were according to the manufacturer’s directions (Bio-Rad, Hercules, CA, USA). Median fluorescence intensity (MFI) was measured using the Luminex 100 system and data analysis using Bio-Plex Manager™ 6.0 software (Bio-Rad, Hercules, CA, USA). The results were then expressed in pg/mL. The intra- and inter-assay CVs (coefficient of variation) were <5.0% and <6.7%, respectively.

### 2.7. Statistical Analysis

Quantitative variables were expressed as mean (SD) or median (inter-quartile range, IQR). A two-tailed *p* value less than 0.05 was considered statistically significant. The Shapiro-Wilk test was applied to evaluate the distribution of variances. The difference between two groups was analyzed by *t*-test or Mann-Whitney Rank Sum test, as appropriate. In addition, Pearson’s or Spearman’s correlation coefficients were performed to identify correlations of blood variables.

## 3. Results

### 3.1. Body Weights and Feed Intake

**Figure 1:**
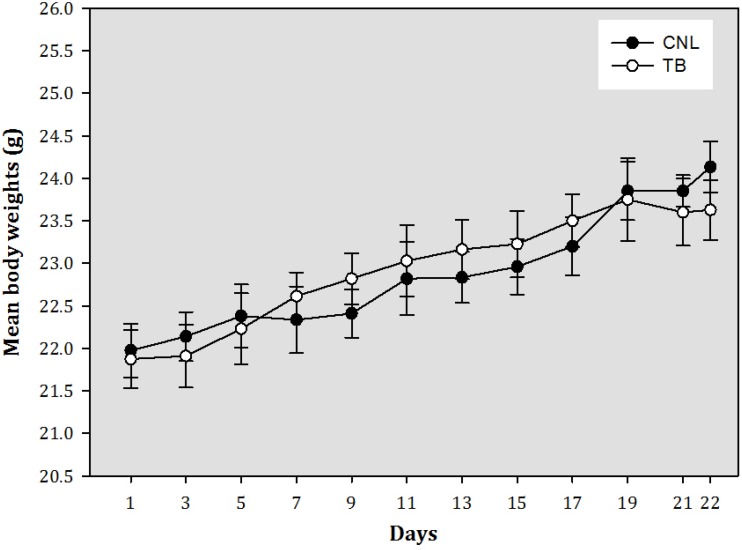
Changes of body weight in mice. CNL = control (*n* = 10); TB = tumor-bearing (*n* = 10). No significant differences were found between CNL and TB groups (*p* > 0.05).

There were no significant differences in body weights (Figure 1) and average daily food intake (Table 1) between both groups throughout the experimental period (*p* > 0.05). The TB mouse showed significant increases in organ weights of spleen and liver, and reduced mammary tissue weights compared to the CNL. In addition, final tumor volume and tumor weight were 2.0 ± 0.5 cm^3^ and 1.5 ± 0.4 g, respectively.

**Table 1:** Daily food intake and organ weights in mice ^1,2^.

	Food intake (g)	Organ weights (g)						
		Liver	Kidney	Brain	Lung	Thymus	Spleen	Mammary
CNL	3.3 ± 0.3	1.02 ± 0.13	0.31 ± 0.03	0.43 ± 0.02	0.13 ± 0.02	0.05 ± 0.01	0.10 ± 0.01	0.14 ± 0.03
TB	3.1 ± 0.3	1.23 ± 0.13 *	0.33 ± 0.02	0.42 ± 0.03	0.13 ± 0.02	0.04 ± 0.01	0.27 ± 0.06 *	0.09 ± 0.03 *

^1^ Values are means ± SD. ^2^ CNL = control (*n* = 10); TB = breast tumor-bearing (*n* = 10). * Values are significantly different compared with CNL (*p* < 0.05).

### 3.2. Blood VEGF, MDA and Se Levels

**Figure 2:**
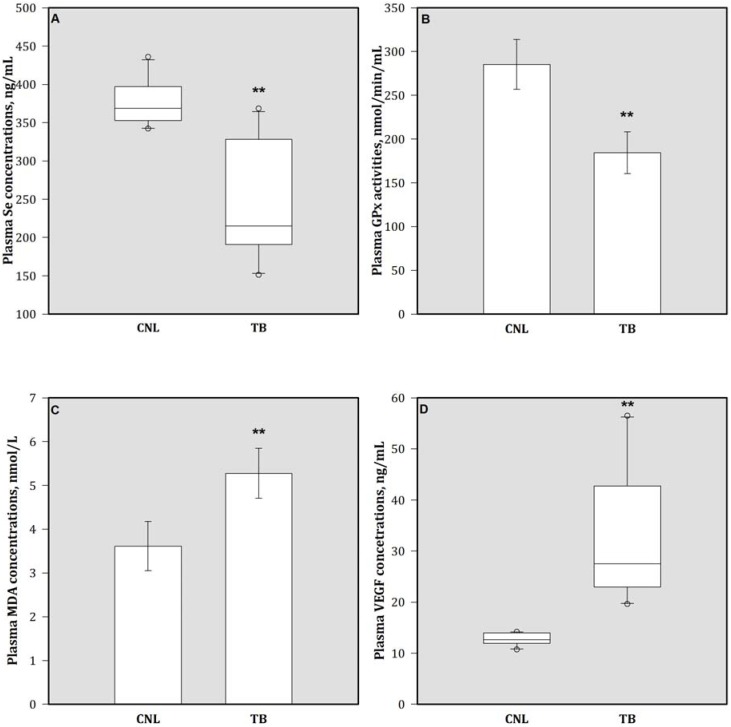
Plasma (**A**) Se, (**B**) GPx activity, (**C**) MDA, and (**D**) VEGF concentrations in mice. Bars are mean (SD) or median (IQR). Values above the box plots are outliers. ** *p* < 0.05 represents comparison with CNL group observation. Se = selenium; GPx = glutathione peroxidase; MDA = malondialdehyde; and VEGF = vascular endothelial growth factor.

There were higher plasma concentrations of VEGF in the TB group than those in the CNL animals (Figure 2). Significant decreases in plasma Se concentrations and GPx activities of TB animals were observed in comparison with CNL animals. On the contrary, the plasma concentrations of MDA in TB group increased significantly compared to the CNL group.

**Figure 3:**
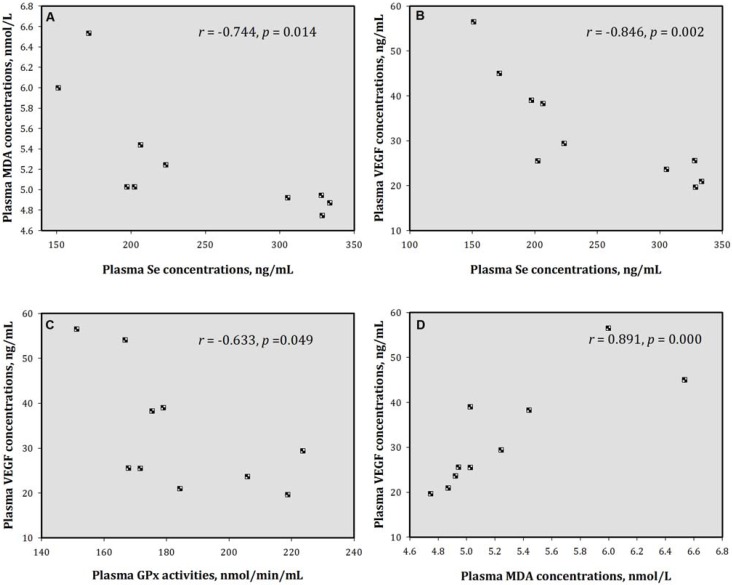
Relationships of the plasma variables in TB mice. Plasma Se concentrations were correlated with (**A**) MDA and (**B**) VEGF concentrations; Plasma VEGF concentrations were correlated with (**C**) Px activity and (**D**) MDA products. Se = selenium; GPx = glutathione peroxidase; MDA = malondialdehyde; and VEGF = vascular endothelial growth factor.

For TB animals, plasma Se concentrations showed significant relationships to plasma MDA (*r* = −0.744) and VEGF (*r* = −0.846) concentrations. Plasma VEGF concentrations were related to GPx activities (*r* = −0.633) and MDA concentrations (*r* = 0.891) (Figure 3). 

### 3.3. Tissue Levels of Se and MDA

**Figure 4:**
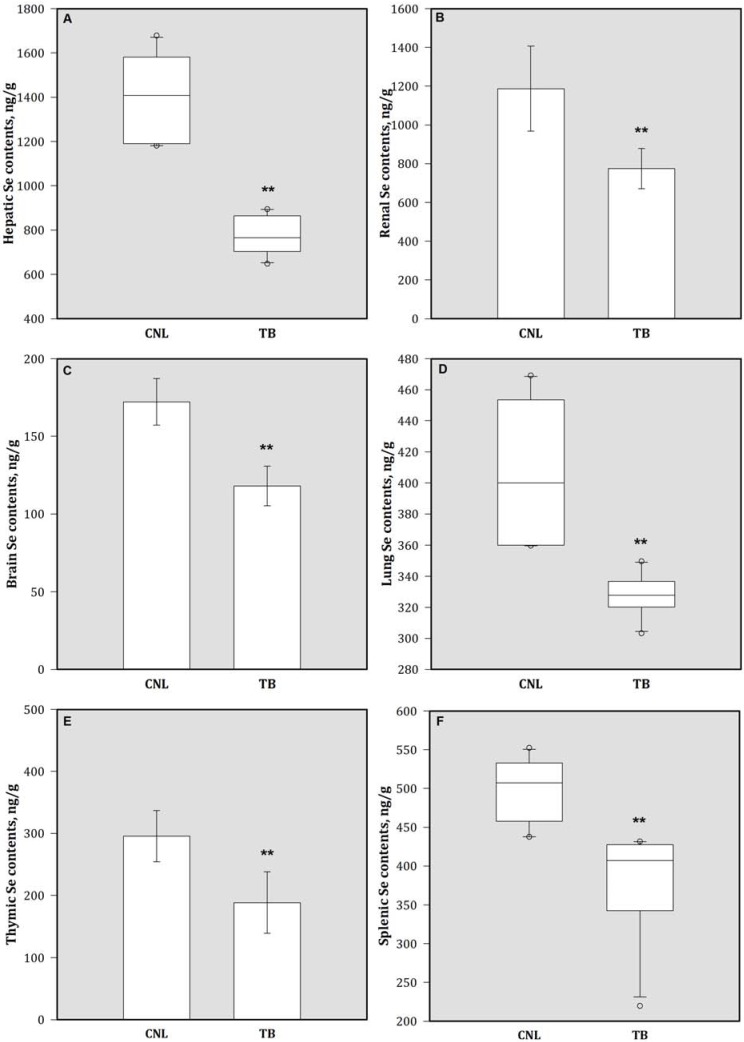
Tissue Se concentrations in (**A**) liver, (**B**) kidney, (**C**) brain, (**D**) lung, (**E**) thymus, and (**F**) spleen. Bars are mean (SD) or median (IQR). Values above the box plots are outliers. ** *p* < 0.05 represents comparison with CNL group observation. Se = selenium.

**Figure 5:**
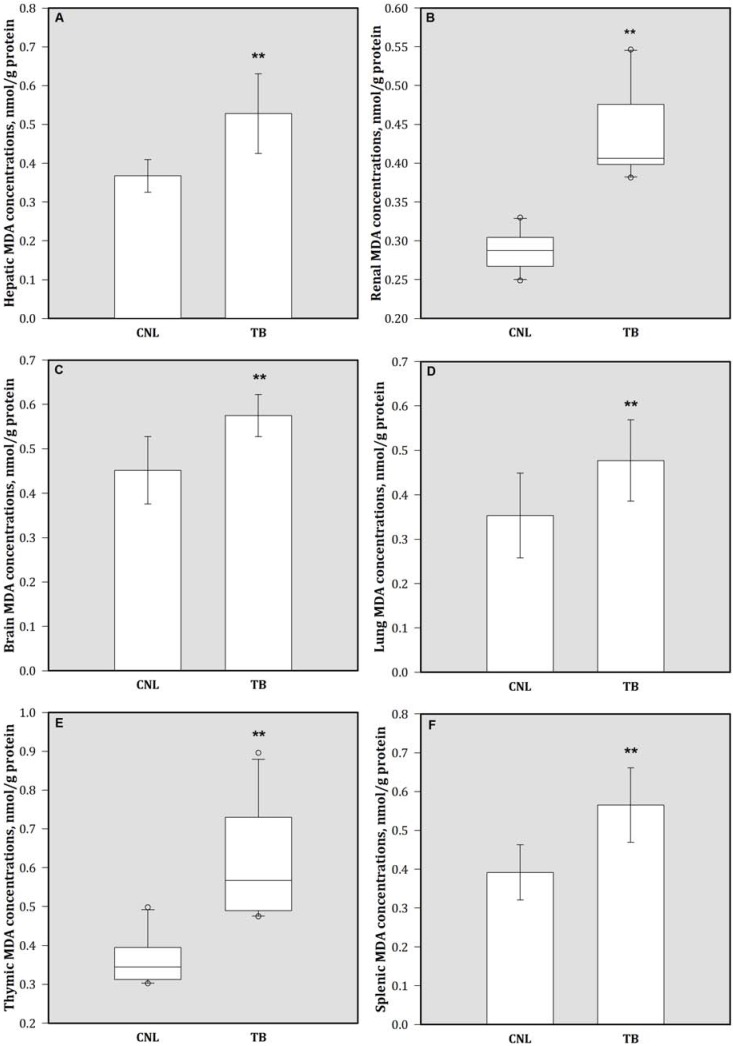
Tissue MDA products in (**A**) liver, (**B**) kidney, (**C**) brain, (**D**) lung, (**E**) thymus, and (**F**) spleen. Bars are mean (SD) or median (IQR). Values above the box plots are outliers. ** *p* < 0.05 represents comparison with CNL group observation. MDA = malondialdehyde.

Compared to CNL, the Se concentrations of different tissues including liver, kidney, brain, lung, spleen, and thymus were decreased significantly in TB animals (Figure 4). By contrast, breast tumor-bearing animals showed increases in MDA concentrations in these tissues compare to the CNL group (Figure 5). There were no significantly differences in mammary tissue Se (149.19 ± 31.03 ng/g *vs.* 165.76 ± 42.99 ng/g) and MDA (0.68 ± 0.25 nmol/g protein *vs.* 0.58 ± 0.20 nmol/g protein) concentrations between CNL and TB animals (data not shown). In addition, TB mice had lower GPx activity in various tissues than CNL (Table 2).

**Table 2:** Antioxidant enzyme GPx activities in various tissues of mice ^1,2^.

	GPx activity (nmol/min/mg protein)			
	Liver	Kidney	Brain	Lung
CNL	381.13 ± 27.22	20.88 ± 4.60	42.13 ± 2.02	40.17 ± 3.34
TB	324.00 ± 20.24 *	13.12 ± 2.76 *	35.21 ± 4.30 *	30.22 ± 5.29 *

^1^ Values are means ± SD. ^2^ CNL = control (*n* = 10); TB = breast tumor-bearing (*n* = 10); * Values are significantly different compared with CNL (*p* < 0.05).

## 4. Discussion

In the present preliminary study, breast tumor-bearing (TB) mice had abnormal distribution of the essential trace mineral Se and a more severe oxidative stress status (as indicated by increased amounts of MDA product and reduced GPx activity) compared to the findings in controls. In addition, decreased plasma Se concentrations were associated with elevated plasma VEGF and increased MDA concentrations in TB mice.

Compared with the findings in control mice, TB mice exhibited non-significantly differences for daily food intake and net body weight to exclude tumor mass, as well as higher hepatic and splenic weights. Reduced food intake can contribute to cachexia, an important predictor of poor outcome and high mortality rate in patients with cancer [27]. As cancer-induced cachexia is a common manifestation, a large number of solid tumors, not including breast cancer, are associated with a higher frequency of cachexia [28]. Furthermore, for patients with hormone-dependent breast cancer, tamoxifen is the major adjuvant treatment. TB mice treated with tamoxifen display lower daily intake [29], and can further reduce the intake of some foods that are high sources of antioxidants. Conversely, the hepatomegaly and splenomegaly occur in TB animals may be a component of the host response to the cancer [30]. Thus, their presence may be linked to cancer cell invasion during tumorigenesis.

Oxidative stress, defined as a disruption between pro-oxidant and antioxidant systems, can result in cellular damage [31]. Increased oxidative stress in breast tumor tissues compared to the levels in non-malignant tissues has been observed [32,33]. The development of cancer results in oxidative stress, which may in turn promote cancer progression [34]. Excess free radicals increase VEGF concentrations, which are involved in the tumor angiogenesis [35]. Elevated plasma VEGF concentrations have been observed in patients with cancer [36,37]; additionally, the overexpression of VEGF and higher plasma VEGF concentrations are associated with metastasis formation and a poor prognosis [38,39]. The present study demonstrated that TB mice have higher plasma MDA products, which were associated with increased plasma concentrations of VEGF. Further, increased MDA contents were observed in various tissues of TB animals may be attributed to antioxidant capacity lost during tumor growth. It appears that tissues are exposed to higher oxidative stress during cancer, thus increasing the risk of tumor cell invasion and metastasis.

Se plays a vital role in antioxidant enzyme GPx, which exerts cancer-preventive effects and anti-tumorgenic activity [40]. Reduced plasma GPx activity and Se concentrations have been found in patients with metastatic cancer [41]. Moreover, reduced GPx activity is inversely related to cancer progression [42]. Se treatment increases GPx expression and reduces the expression of VEGF in tumor cells [43]. Our results support previous studies revealing a negative correlation observed between plasma Se status and VEGF concentrations during tumor promotion [44]. 

Relative to controls, these TB animals have reduced concentrations of Se in plasma and various tissues including the liver, kidney, spleen, brain, lung, and thymus. The decreases in Se status may be due, in part, to a reduction in antioxidant capacity thus increased those tissues’ susceptibility to oxidative damage. Protection against tumor growth may also contribute to increased tumor Se uptake [45], thus lowering Se status in those tissues. The possible causes have not, however, been elucidated. By contrast, mammary tissue Se was not lower in TB mice; one possible explanation for this result is that decreased mammary fat pad contents were observed compared with CNL animals. It appears that alterations in Se concentrations occur during cancer development [23]; whereas improved Se status associated with decreased oxidative stress may be useful for preventing the progression of breast cancer [46].

## 5. Conclusion

The present preliminary study found that breast tumor-bearing mice had lower Se concentrations and GPx activities in plasma and various tissues, which may have contributed to the elevation of oxidative stress and amplification of VEGF. Disrupted homeostasis of Se is thus a potential risk factor for progression of breast tumor and metastasis. Therefore, Se supplementation may be needed for the maintenance of Se homeostasis that is beneficial to patients with breast cancer.
